# The Effect of Water Co-Feeding on the Catalytic Performance of Zn/HZSM-5 in Ethylene Aromatization Reactions

**DOI:** 10.3390/ijms25042387

**Published:** 2024-02-17

**Authors:** Jiabei Shao, Pengcheng Feng, Baichao Li, Jie Gao, Yanyan Chen, Mei Dong, Zhangfeng Qin, Weibin Fan, Jianguo Wang

**Affiliations:** 1 State Key Laboratory of Coal Conversion, Institute of Coal Chemistry, Chinese Academy of Sciences, P.O. Box 165, Taiyuan 030001, China; 2 University of Chinese Academy of Sciences, Beijing 100049, China

**Keywords:** ethylene aromatization reaction, Zn/HZSM-5 catalyst, water co-feeding, dehydrogenation reaction

## Abstract

During the methanol-to-aromatics (MTA) process, a large amount of water is generated, while the influence and mechanism of water on the activity and selectivity of the light olefin aromatization reaction are still unclear. Therefore, a study was conducted to systematically investigate the effects of water on the reactivity and the product distribution in ethylene aromatization using infrared spectroscopy (IR), intelligent gravitation analyzer (IGA), and X-ray absorption fine structure (XAFS) characterizations. The results demonstrated that the presence of water reduced ethylene conversion and aromatic selectivity while increasing hydrogen selectivity at the same contact time. This indicated that water had an effect on the reaction pathway by promoting the dehydrogenation reaction and suppressing the hydrogen transfer reaction. A detailed analysis using linear combination fitting (LCF) of Zn *K*-edge X-ray absorption near-edge spectroscopy (XANES) on Zn/HZSM-5 catalysts showed significant variations in the state of existence and the distribution of Zn species on the deactivated catalysts, depending on different reaction atmospheres and water contents. The presence of water strongly hindered the conversion of ZnOH^+^ species, which served as the active centers for the dehydrogenation reaction, to ZnO on the catalyst. As a result, the dehydrogenation activity remained high in the presence of water. This study using IR and IGA techniques revealed that water on the Zn/HZSM-5 catalyst inhibited the adsorption of ethylene on the zeolite, resulting in a noticeable decrease in ethylene conversion and a decrease in aromatic selectivity. These findings contribute to a deeper understanding of the aromatization reaction process and provide data support for the design of efficient aromatization catalysts.

## 1. Introduction

The methanol-to-aromatics (MTA) process, as an important non-petroleum conversion route to produce aromatics, is of great strategic significance for optimizing the utilization of carbon-containing resources such as coal, natural gas, and biomass and alleviating petroleum shortages [1,2]. In the MTA reaction process, it is generally believed that the methanol conversion route involves the dehydration of methanol on the catalyst, followed by the formation of olefins via a direct or indirect mechanism, and then further conversion of olefins to aromatics [3]. In all proposed methanol conversion mechanisms [4,5], water is considered a primary reaction byproduct, and its presence is often overlooked in terms of its impact on the catalyst and the methanol conversion process.

The methanol-to-aromatics process includes methanol dehydration, methanol-to-light olefin conversion, and light olefin aromatization. Therefore, the presence of water may have a considerable influence on the aromatization reaction in both the methanol-to-olefin (MTO) reaction and the aromatization reaction of light olefins. This has been noted in a few reports in the literature. Studies have shown [6,7] that water co-feeding in the MTO reaction process can prolong the methanol conversion induction period, increase the olefin selectivity, and delay catalyst deactivation. Researchers believe that this is because methanol, olefins, and water compete for adsorption at the acidic sites of the catalyst, and the active sites have a stronger adsorption capacity for water, reducing the secondary reaction of olefins and slowing down the rate of carbonaceous species formation. The beneficial effects are usually attributed to the stronger adsorption of water at the active site, which reduces secondary reactions of the desired hydrocarbon products from taking place. Interestingly, water molecules have been found to not only partially facilitate the conversion of hydrophilic molecules such as methanol, ethanol [8], and propanol [9] but also significantly impact the activation of hydrophobic molecules such as isobutane. Chen et al. [10] discovered that the presence of a small amount of water (<1 water molecule per active site) significantly increased the rate of alkane C-H activation in isobutane, while a high water content (2–3 water molecules per active site) slowed down the reaction rate. These results indicate that water can also be an active participant in reactions involving hydrophobic molecules in solid acid catalysts, possibly via transition state stabilization, as long as the water concentration is essentially stoichiometric.

Light olefin aromatization is the main pathway for aromatic hydrocarbon generation in the MTA process. In order to obtain a high-performance MTA catalyst, it is necessary to improve its activity in the light olefins’ aromatization reaction. However, in the case of light olefin aromatization, the water effect is rather complex according to the complexity of the formation of aromatics via the oligomerization, cyclization, dehydrogenation, and hydrogen transfer reaction [11,12,13]. In their study on the effect of water on the performance of HZSM-5 catalyst in the aromatization reaction of ethylene, Wang et al. [14] found that water preferentially adsorbs on the Brønsted acid sites of HZSM-5, forming (Z-OH...H_2_O) hydrogen-bonded complexes and H^+^(H_2_O)_n_ species. These species are located inside the zeolite channels, preventing the oligomerization of ethylene, alkene-induced hydrogen transfer reactions, and the progression of the hydrocarbon pool mechanism inside the channels. They also observed that when most of the water desorbs from the Brønsted acid sites, the conversion of ethylene recovers. This is because physically adsorbed water enhances the channel effect of the catalyst and changes the operating mechanism of HCP on HZSM-5 molecular sieves at high temperatures.

Zn/HZSM-5 possesses both acidic and dehydrogenation active sites, which results in a distinctive shape-selective effect and remarkable activity in aromatization reactions [15,16,17,18,19]. Due to the dehydrogenation reactions that occur during aromatization, the reaction is carried out at high temperatures in a H_2_ atmosphere, resulting in the dynamic change of the state and distribution of Zn species. Triwahyono et al. [20] found that Zn^2+^ species located in ion-exchange sites are difficult to completely reduce to metallic Zn^0^ even at high temperatures of 900 °C. Biscardi et al. [21] found that under a hydrogen atmosphere, Zn^2+^ species in Zn/HZSM-5 zeolite are not easily reduced to metallic Zn due to the confinement effect of the zeolite, while ZnO species have a weak interaction with the catalyst and are easily reduced to metallic Zn and evaporate in the form of steam. Gao et al. [22] found that H_2_ pretreatment can activate the Zn/HZSM-5 catalyst, significantly increasing the activity of the ethylene aromatization reaction. However, when the pretreatment temperature is above 600 °C, large ZnO species in the zeolite are reduced and sublime, resulting in the loss of Zn in the catalyst. Geng et al. [23] found that the main Zn species present at different stages of the ethylene aromatization reaction include ZnOH^+^, ZnO clusters, ZnO particles, and metallic Zn species. The composition of these species undergoes significant changes throughout the reaction, with ZnO clusters migrating to the external surface of the zeolite, forming ZnO particles, and then being reduced and sublime. However, there is a lack of research on the influence of water on the aromatization reaction behavior of Zn/HZSM-5 and the composition of Zn species during the reaction process.

In the process of producing aromatics from methanol and light olefins, the presence of water is often unavoidable, and it greatly influences the structural stability of zeolite catalysts, the state of metallic active centers, and the catalytic mechanism. Therefore, this paper uses the ethylene aromatization reaction as a probe to study the influence of water on the catalytic activity centers and catalytic mechanism of the Zn/HZSM-5 zeolite catalyst during the aromatization reaction in order to provide data support for the design of efficient MTA catalyst.

## 2. Results and Discussion

### 2.1. Comparison of H_2_O and N_2_ Atmosphere on the Performance of Zn/HZSM-5 in ETA

**Figure 1:**
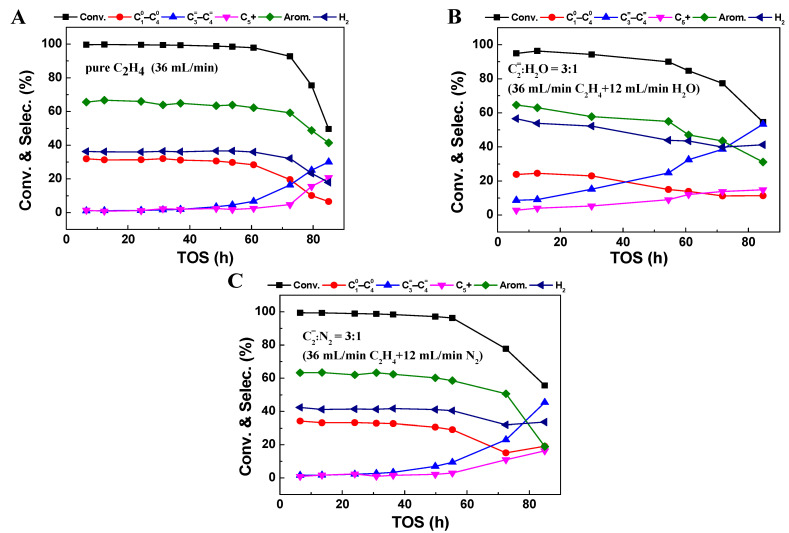
The conversion and product selectivity at different reaction times of ethylene aromatization reaction with the feed of (**A**) pure C_2_H_4_, (**B**) C_2_^=^:H_2_O = 3:1 (75%C_2_H_4_–25% H_2_O), and (**C**) C_2_^=^:N_2_ = 3:1 (75%C_2_H_4_–25%N_2_) over Zn/HZSM-5. Reaction conditions: 470 °C, 0.1 MPa, WHSV = 1.8 h^−1^.

Figure 1 depicts the changes in the ethylene conversion and product selectivity with time on stream (TOS) over Zn/HZSM-5 catalyst under different atmospheres (pure ethylene feed, ethylene–water co-feed, and ethylene–nitrogen co-feed). Regardless of the atmosphere, the ethylene aromatization products on the Zn/HZSM-5 catalyst mainly consist of aromatics dominated by benzene, toluene, and xylene (BTX); light olefins (C_3_^=^~C_4_^=^); light alkanes (C_1_^0^~C_4_^0^); and C_5_+ hydrocarbons; as well as hydrogen produced from the dehydrogenation reaction. However, atmospheric conditions significantly influence the conversion, product distribution, and stability.

In the case of 100% ethylene feed (Figure 1A), the ethylene conversion on the Zn/HZSM-5 catalyst reaches over 99.6%, with a selectivity toward aromatics of 66.6% and a stability of nearly 60 h. When ethylene is co-fed with either water or nitrogen gas (Figure 1B,C), although the reaction behavior and product selectivity trends with TOS are similar to that in pure ethylene, the presence of water and nitrogen gas exerts different influences. It seems that the N_2_ atmosphere has little influence on the ethylene conversion, while the selectivity toward aromatics decreases slightly to 62%, and the stability decreases to approximately 50 h. However, under a H_2_O atmosphere, the ethylene conversion decreases considerably, with a maximum conversion of only 95.9%. The selectivity toward light alkanes (24.5%) and aromatics (63.0%) also decreases, while the selectivity toward olefins (9.1%) increases significantly. Notably, the selectivity toward hydrogen gas in the presence of water (49.6%) is significantly higher than in pure ethylene (36.1%) or nitrogen atmospheres (42.3%), suggesting a potential change in the pathway of aromatics formation under these conditions (Figure 1B).

**Figure 2:**
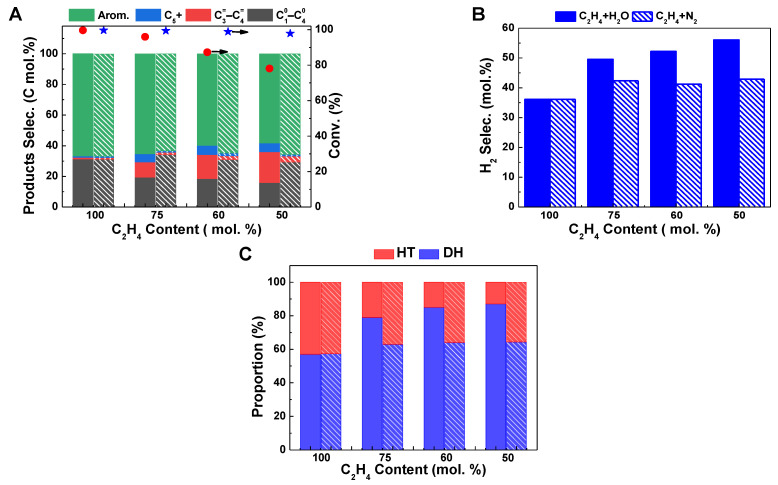
Comparison of ethylene aromatization reaction on Zn/HZSM-5 with the presence of H_2_O (filled bars) and N_2_ (sparsely filled bars). (**A**) The ethylene conversion (red circle: C_2_H_4_; blue star: C_2_H_4_+H_2_O) and product selectivity, (**B**) the H_2_ selectivity, and (**C**) the proportion of hydrogen transfer pathway and dehydrogenation pathway in ethylene aromatization reaction in H_2_O or N_2_ atmospheres with different C_2_H_4_ contents. Reaction conditions: 470 °C, 0.1 MPa, WHSV = 1.8 h^−1^, TOS of 12 h.

The impact is more significant with a further increase in the water content in the feed (Figure 2). By increasing the relative proportion of H_2_O to 50% without changing the ethylene feeding rate, the ethylene conversion and aromatic selectivity dropped to 78.1% and 58.1%, respectively, accompanied by a further increase in hydrogen selectivity to 56.1% (Figure 2A,B). This indicates that the presence of water inhibits the conversion of ethylene and the formation of aromatics but accelerates the dehydrogenation reaction. However, the effects of co-feeding N_2_ gas on ethylene conversion and product selectivity are not as significant as those observed in a water atmosphere. When the relative proportion of N_2_ increases to 50%, the ethylene conversion is still maintained at 97.9%, maintaining the aromatic selectivity of 65.6% and hydrogen selectivity of 42.9%. Comparing the changes in ethylene aromatization reaction behavior in the presence of the same proportion of H_2_O and N_2_, it is shown that the change in ethylene contact time on the Zn/HZSM-5 catalyst does indeed affect reaction stability, but it is not the main factor leading to changes in product distribution when water and ethylene are co-fed. Therefore, the changes in ethylene reaction behavior, especially changes in product distribution, in the presence of water may be due to the altered ethylene adsorption behavior on the catalyst or changes in the state of active sites on the catalyst.

### 2.2. Analysis of the Formation of Aromatics over HZSM-5 and Zn/HZSM-5

**Scheme 1:**
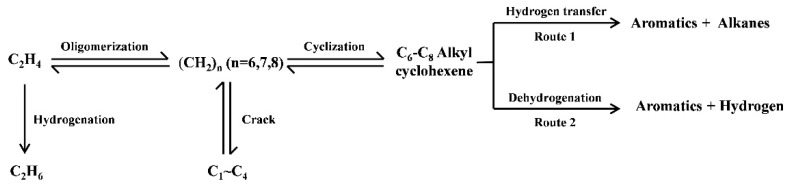
The pathways for aromatics formation in the ethylene aromatization process.

It is generally believed that the formation of aromatic hydrocarbons can be achieved through two pathways: the hydrogen transfer (HT) reaction between cycloalkenes and light alkenes to produce aromatic hydrocarbons and light alkanes or the dehydrogenation (DH) of cycloalkene molecules to produce aromatic hydrocarbons and hydrogen (Scheme 1) [12,24]. Therefore, based on the selectivity of aromatic hydrocarbons, hydrogen gas, and light alkanes in the product, the relative proportions of the dehydrogenation pathway and the hydrogen transfer pathway in the ethylene aromatization reaction were calculated, as shown in Figure 2C. It can be seen that with the increase in the proportion of H_2_O in the reactants, the proportion of the dehydrogenation reaction significantly increases, while the proportion of the hydrogen transfer reaction decreases on the Zn/HZSM-5 molecular sieve. However, in the presence of N_2_, the relative proportions of the dehydrogenation reaction and the hydrogen transfer reaction are basically fixed and do not change with the variation of the composition of the reactants (Figure 2C). This further indicates that in this process, H_2_O may affect the composition of the active centers in the Zn/HZSM-5 catalyst and promote the occurrence of the dehydrogenation reaction.

**Figure 3:**
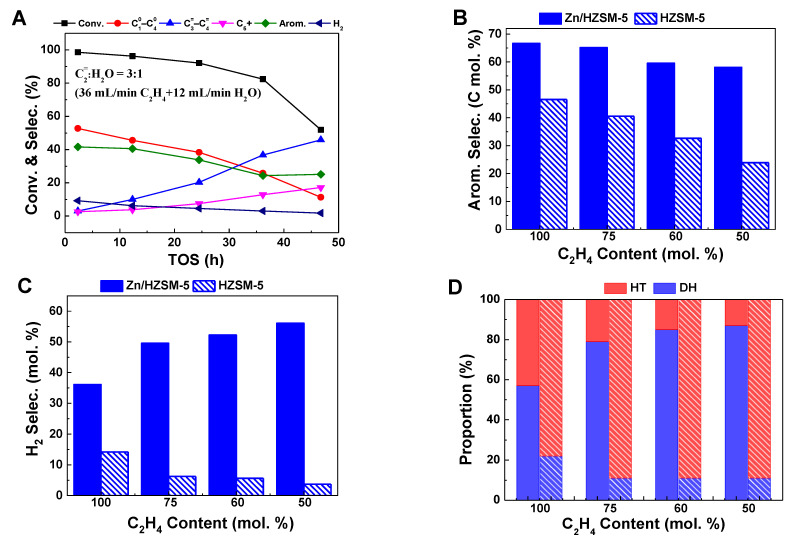
The ethylene conversion and product selectivity at different reaction times of ethylene aromatization reaction on HZSM-5 (**A**) and comparison of the aromatic selectivity (**B**), H_2_ selectivity (**C**), and proportion of hydrogen transfer pathway and dehydrogenation pathway (**D**) on Zn/HZSM-5 (filled bars) and HZSM-5 (sparsely filled bars). Reaction conditions: 470 °C, 0.1MPa, WHSV = 1.8 h^−1^, TOS of 12 h.

To make a clearer comparison of the influence of water on HZSM-5 and ZnZSM-5 catalysts, the above-mentioned data were further summarized in Table 1. It can be seen that, on the HZSM-5 zeolite, the influence of water does not seem to have a significant effect on the selectivity of hydrogen. A similar report by Wang et al. [12] also shows that, on the HZSM-5 zeolite, although the presence of water significantly changes the conversion of ethylene and the selectivity of products, the promotion effect on the generation of H_2_ is neglectable. This may be related to the use of a HZSM-5 catalyst rather than a Zn/HZSM-5 catalyst. In the Zn/HZSM-5 zeolite, the ZnOH^+^ species formed by the interaction of zinc species with the Brønsted acid site is considered the active site for dehydrogenation [25]. At the same time, the Brønsted acid center in the HZSM-5 zeolite is also responsible for the dehydrogenation reaction. Therefore, the change in the relative distribution of the dehydrogenation reaction and hydrogen transfer reaction infers that co-feeding H_2_O may affect the content of dehydrogenation active sites on the catalyst. In order to further explore the influence of H_2_O on the catalyst, the catalytic performance of HZSM-5 zeolite in ethylene co-feeding systems with different water contents was studied (Figure 3A). It can be observed that when water and ethylene are co-fed for aromatization reaction on HZSM-5, the conversion and selectivity of aromatic hydrocarbons decrease with increasing water content, similar to those observed on Zn/HZSM-5. Wang et al. [14] found the same results (Table 1) when studying the effect of water on ethylene aromatization reaction using a HZSM-5 catalyst. They believed that when water is co-fed, a hydrogen-bonded complex (Zeo-OH⋯H_2_O) is formed from the adsorbed H_2_O and the Brønsted acid site, competing with ethylene for adsorption, thus inhibiting the occurrence of aromatization reaction. However, it is worth noting that with an increase in water content in the feed, the H_2_ selectivity on the HZSM-5 catalyst significantly decreases, and the proportion of the dehydrogenation pathway decreases with increasing water content (Figure 3B–D). This is in contrast to the change in H_2_ generation on Zn/HZSM-5, indicating that the presence of water may promote the formation of the dehydrogenation active species ZnOH^+^. To elucidate the reasons for the influence of water on the ethylene aromatization reaction, research was conducted on the effect of water on the existence state of the active centers of Zn species.

### 2.3. Effect of the Presence of H_2_O on the Zn Species

It is widely recognized that the Zn species in zeolites are highly complex and may include ZnO particles, ZnO nanocrystals, ZnO clusters, ZnOH^+^, and Zn^2+^ with the structure of O-Zn^2+^-O or [Zn-O-Zn]^2+^ [26]. Due to the high dispersion of Zn species in the zeolite, accurately characterizing and determining their composition and existence state poses a significant challenge. XAFS technique is an effective method for characterizing the local geometry and providing detailed information about the fine structure of Zn species. XAFS data analysis includes X-ray absorption near-edge structure (XANES) and extended X-ray absorption fine structure (EXAFS) methods [23]. EXAFS can selectively observe the specific chemical composition of Zn and detect its site environment based on bond length and coordination number. On the other hand, XANES is sensitive to the local structure of the metal ion and can quantitatively analyze the specific compositional forms of standard compounds [27,28]. In the previous work, based on the characteristics of XAFS, we have established a method to accurately analyze the distribution of Zn species in zeolites using LCF analysis of XANES spectra [29]. In order to investigate the effect of water on the ethylene aromatization reaction, we characterized the fresh catalyst and deactivated Zn/HZSM-5 catalysts used in the ETA reaction under different atmospheres.

**Figure 4:**
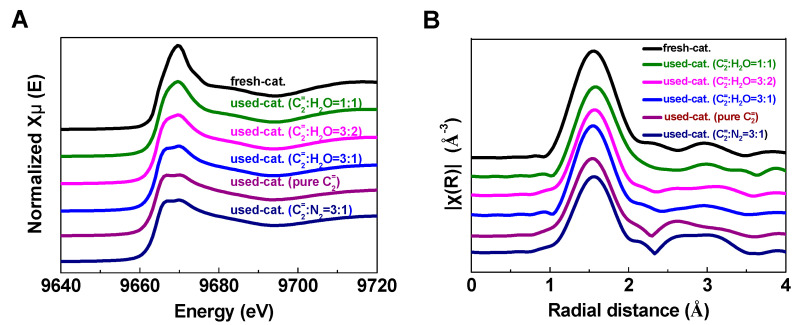
Zn *K*-edge XANES (**A**) and EXAFS spectra (**B**) of Zn/HZSM-5 fresh catalyst and deactivated Zn/HZSM-5 catalysts used in ETA reaction with different atmospheres.

Figure 4 shows the background-subtracted and normalized XANES at the Zn *K*-edge and the plots of the Fourier transforms of the EXAFS spectra (k^2^ weighted over k range from 2.3 to 11.7 Å^−1^) of the fresh and used Zn/HZSM-5 catalysts under different atmospheres. It is obvious that the Zn *K*-edge absorption peak, the white line peak centered at 9669 eV, varies in shape and intensity on different catalysts. The absorption peak of the fresh catalyst is sharper, while the reacted catalysts show significant splitting and broadening of the absorption edge peak. This indicates that the state of Zn species in the fresh catalyst is closer to the ionic state, while the reacted catalysts show more covalent bonding characteristics. It is worth noting that with the increase in water content in the reaction system, the proportion of ionic state of Zn in the reacted catalysts is higher, such as the absorption peak of the catalyst (used Zn/HZSM-5, C_2_^=^:H_2_O = 1:1) is close to that of the fresh catalyst.

The variation of E_0_ values illustrates the same issue (Table 2). Compared to fresh catalysts, the E_0_ values of deactivated catalysts shift toward lower energies, indicating a decrease in the electron density and valence state of Zn. Similar phenomena were observed by Geng et al. [23] during the investigation of the migration of Zn species on Zn/HZSM-5 catalysts in the ethylene aromatization reaction. As the reaction proceeds, both the coordination environment and oxidation state of Zn species change. In fresh catalysts and deactivated catalysts, the dominant species are ZnOH^+^ and ZnO clusters, respectively, resulting in a shift of E_0_ value to lower energy with the reaction process. It is interesting that in the presence of water, the changes in electron density and oxidation state of Zn in deactivated catalysts are significantly suppressed, most notably in the reaction system with the highest water content (used Zn/HZSM-5, C_2_^=^:H_2_O = 1:1) where the E_0_ value of the catalyst is almost identical to that of the fresh catalyst (9664.43 eV). This indicates that the presence of water slows down the transition of Zn species.

The Zn-O coordination parameters obtained from the EXAFS fitting (Figure 4B) show that the coordination number (CN) of the fresh Zn/HZSM-5 catalyst is 6.49, suggesting the octahedral coordination structure of Zn in zeolite. This may be attributed to the presence of ZnOH^+^, which is formed from the interaction of the Zn species with the Brønsted acid in the zeolite [30]. However, the CN of O atoms around Zn atoms on the deactivated catalysts significantly decreases, indicating a transition from a 6-fold to a 4-fold coordination structure of Zn species on the catalyst. Nevertheless, the trend of decreasing coordination number of Zn-O structure in the deactivated catalysts is significantly slowed down with increasing water content in the ethylene reaction, with the used Zn/HZSM-5 (C_2_^=^:H_2_O = 1:1) catalyst having a coordination number of 6.09 and a coordination distance of 2.03, which is close to that of the fresh catalyst.

To further determine the distribution of Zn species on different catalysts, the LCF analysis on the Zn *K*-edge XANES spectra of the catalysts was performed. To accurately describe the structure of Zn species, several typical Zn compounds are chosen as reference samples to represent the potential structure of Zn species that may exist. The identified Zn species in the present work include ZnO particles with a grain size of approximately 50 nm, Zn/HZSM-5-IE-0.66% representing ZnOH^+^ species, Zn/silicalite-1–0.1% representing confined ZnO clusters in pore channels, Zn/silicalite-1–5% representing crystalline ZnO attached to the outer surface, and Willemite referred to as Zn-O-Si structure [23].

**Figure 5:**
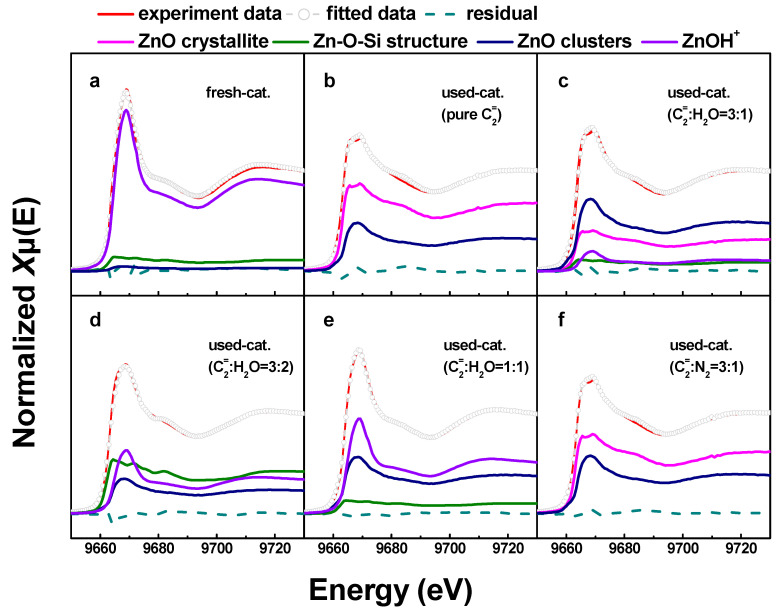
Comparison between experimental (solid red lines) and LCF analysis (circles) of the XANES collected for the Zn/HZSM-5 samples of (**a**) fresh Zn/HZSM-5, (**b**) Zn/HZSM-5 used in pure ethylene, (**c**) Zn/HZSM-5 used in C_2_^=^:H_2_O = 3:1, (**d**) Zn/HZSM-5 used in C_2_^=^:H_2_O = 3:2, (**e**) Zn/HZSM-5 used in C_2_^=^:H_2_O = 1:1, and (**f**) Zn/HZSM-5 used in C_2_^=^:N_2_ = 3:1.

**Figure 6:**
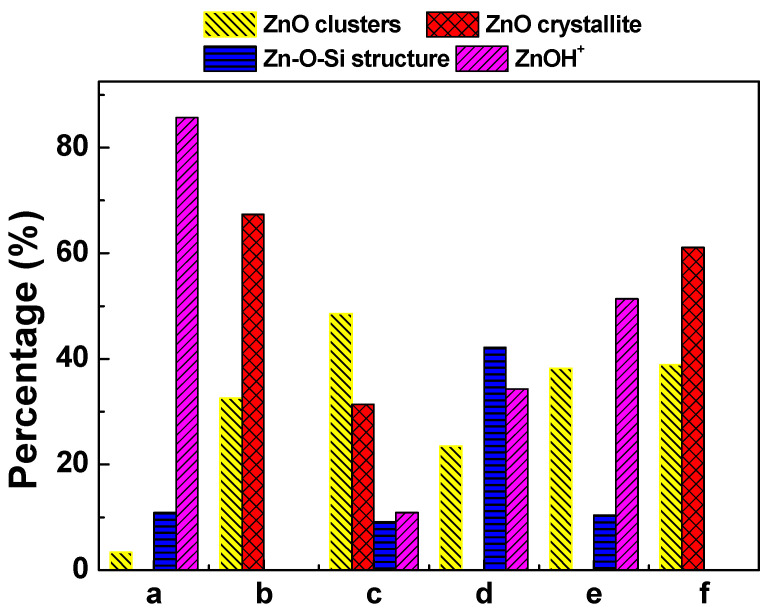
Relative distribution of different Zn species in Zn/HZSM-5 samples of (**a**) fresh Zn/HZSM-5, (**b**) Zn/HZSM-5 used in pure ethylene, (**c**) Zn/HZSM-5 used in C_2_^=^:H_2_O = 3:1, (**d**) Zn/HZSM-5 used in C_2_^=^:H_2_O = 3:2, (**e**) Zn/HZSM-5 used in C_2_^=^:H_2_O = 1:1, and (**f**) Zn/HZSM-5 used in C_2_^=^:N_2_ = 3:1.

Figure 5 shows the LCF analysis of the XANES spectra of fresh and deactivated Zn/HZSM-5 catalysts after ethylene aromatization under different atmospheres. The relative content of the fitted Zn species is shown in Figure 6. In the fresh catalyst, Zn species composed of multi characteristics from ZnOH^+^, Zn-O-Si structure, and ZnO clusters were detected with relative contributions of 85.7%, 10.9%, and 3.4%, respectively. This result confirms the findings from the EXAFS analysis, which suggests that the Zn species in the fresh catalyst mainly consist of octahedral coordinated Zn cations (Table 2). However, the change in the reaction atmosphere significantly affects the existence state and structural distribution of Zn on the deactivated catalysts. In the reaction with pure ethylene or a certain amount of N_2_ gas, the deactivated Zn/HZSM-5 catalyst consists of crystalline ZnO and ZnO clusters with a changing composition depending on the velocity of ethylene. Nevertheless, the disappearance of ZnOH^+^ species in these samples may explain the weak catalytic performance in the dehydrogenation reaction (Figure 6). Similar observations were made by Geng et al. [23] during their study on the migration of Zn species in the ethylene reaction process, where ZnOH^+^ species were observed to transform into ZnO clusters and ZnO crystals. The latter can easily be reduced to metallic Zn and sublime, resulting in the loss of Zn species in the H_2_ atmosphere. However, when water is co-fed with ethylene, an increase in water content leads to an increase in ZnOH^+^ species and a decrease in ZnO content on the deactivated Zn/HZSM-5 catalysts. The concentration of ZnOH^+^ species on deactivated Zn/HZSM-5 catalysts varies from 0% to 10.9%, 34.3%, and 51.4% when used in pure ethylene, C_2_^=^:H_2_O of 3:1, C_2_^=^:H_2_O of 3:2, and C_2_^=^:H_2_O of 1:1 atmospheres, respectively. This indicates that the presence of water in the reaction atmosphere helps to maintain the ZnOH^+^ active site of the catalyst. On the contrary, the proportion of ZnO clusters and crystalline ZnO on deactivated Zn/HZSM-5 catalysts used in a water atmosphere significantly decreases, which suggests that the presence of water during the reaction inhibits the transformation of ZnOH^+^ to ZnO clusters, ZnO crystals, and other structural species, thereby maintaining a higher concentration of ZnOH^+^ species. It is widely known that the ZnOH^+^ species is responsible for the dehydrogenation reaction of alkanes and alkenes [23]. In the ethylene aromatization reaction, the ZnOH^+^ species may serve as active sites for the dehydrogenation of C_6_~C_8_ alkyl cyclohexene, promoting the formation of aromatics and the generation of hydrogen (Scheme 1). Therefore, the higher concentration of ZnOH^+^ species on the deactivated Zn/HZSM-5 catalysts in the reaction system likely contributes to their superior dehydrogenation activity during ethylene aromatization.

### 2.4. Effect of H_2_O Adsorption on ETA

**Figure 7:**
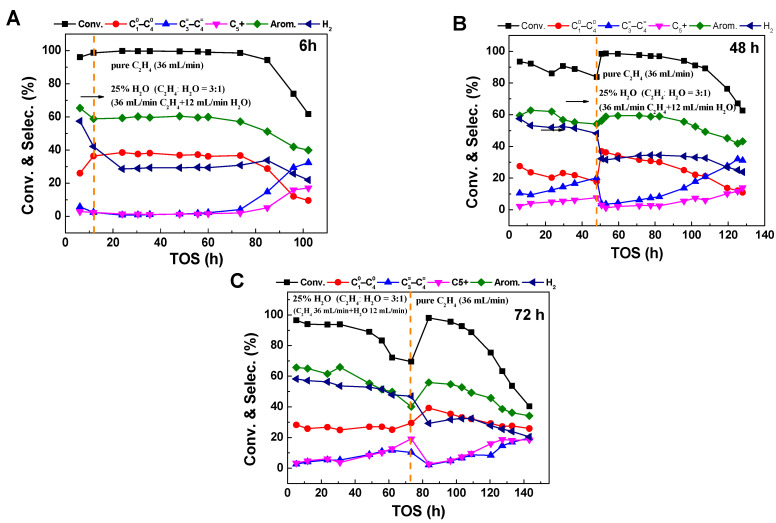
The conversion and product selectivity at different reaction times of ethylene aromatization reaction of ethylene aromatization reaction with switching off 25% water content at reaction times of (**A**) 6 h, (**B**) 48 h, and (**C**) 72 h, further denoted by the orange dashed line. Reaction conditions: 470 °C, 0.1 MPa, WHSV = 1.8 h^−1^.

While the precise characterization of Zn species explains the increase in dehydrogenation ratio in the presence of water to some extent, the reason for the decrease in ethylene conversion remains unclear. In order to investigate the effect of water on the ethylene reaction, comparative experiments were conducted in different reaction stages with water switching. In the reaction with an atmosphere of ethylene to water ratio of 3, the water feed was terminated after 6 h, 48 h, and 72 h of reaction. The ethylene conversion and product distribution can be seen in Figure 7.

It can be seen that in different stages of the reaction, switching off the water feed results in a significant recovery of the ethylene conversion and aromatic selectivity. Particularly, after 72 h of reaction, when the ethylene conversion decreases to about 70%, the removal of water leads to a rapid recovery of the conversion to 99%. This indicates that the main reason for the decrease in ethylene conversion is that water occupies the active sites, making it difficult for ethylene to effectively contact the active centers and resulting in a lower conversion. Removing water restores the active sites, promoting efficient ethylene conversion. Interestingly, although the stability of the ethylene aromatization reaction is poor in a water atmosphere (Figure 1B), cutting off the water feed after a certain period of time in a water atmosphere not only leads to the recovery of reaction activity but also significantly increases the stability of the catalyst.

**Figure 8:**
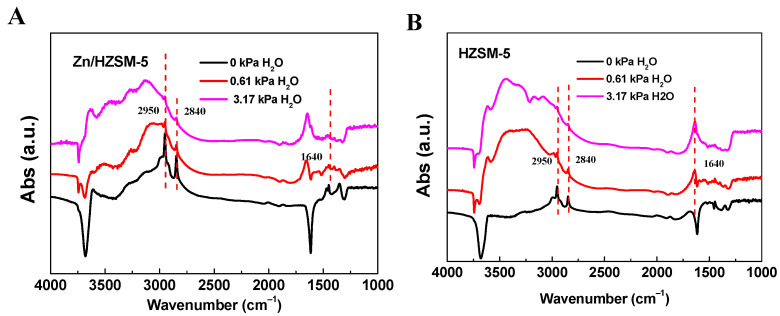
The FT-IR spectra of (**A**) Zn/HZSM-5 and (**B**) HZSM-5 samples with H_2_O and ethylene absorption.

In order to verify the effect of the co-adsorption of water on the ethylene reaction, IR studies of ethylene and water adsorption were conducted (Figure 8). It can be seen that when only ethylene is adsorbed (0 kPa H_2_O), the IR spectra of Zn/HZSM-5 and HZSM-5 samples show symmetric and asymmetric stretching vibration peaks belonging to the CH_2_ groups of ethylene at 2950 and 2840 cm^−1^, respectively [31,32]. When ethylene and water are co-adsorbed, the absorption vibration peak of ethylene decreases, and a bending vibration peak belonging to water appears at 1640 cm^−1^ [33,34]. Furthermore, as the water content further increases, the intensity of the ethylene vibration peak decreases while the intensity of the water vibration peak increases. This further indicates that water competes with ethylene for adsorption, which is the main reason for the decrease in ethylene conversion.

**Figure 9:**
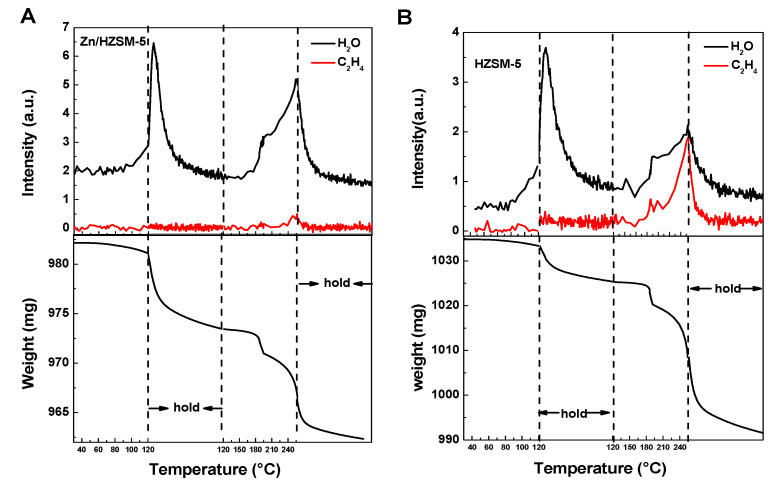
The IGA profiles of H_2_O and ethylene desorption process on (**A**) Zn/HZSM-5 and (**B**) HZSM-5 samples detected using mass spectrometry (upper) and microbalance (bottom).

The comparative adsorption of water and ethylene on HZSM-5 and Zn/HZSM-5 molecular sieves was analyzed by IGA characterization (Figure 9). The IGA profile suggests that, as the desorption temperature elevates from 50 °C to 250 °C with a half-hour dwell at 120 °C, changes in catalysts’ masses are observed at 120 °C and 250 °C. The mass spectrometry analysis indicates that the decrease in weight at 120 °C is attributed to water desorption, while the decrease in weight at 250 °C corresponds to the co-contributions of water and ethylene desorption. For water desorption, the two desorption peaks at 120 °C and 250 °C correspond to physically adsorbed water and chemically adsorbed water, respectively [34]. However, only chemically adsorptive ethylene can be identified on the zeolite samples. The fact that water and ethylene have the same desorption temperature of 250 °C suggests that water and ethylene have similar chemical adsorption strengths on these two catalysts, indicating competitive adsorption between them.

The relative adsorption amounts of water and ethylene on the HZSM-5 catalyst and Zn/HZSM-5 catalyst were calculated based on mass spectrometry signals (Table 3). According to weight analysis, the weight loss on the HZSM-5 catalyst (33.79 mg) is significantly higher than on the Zn/HZSM-5 catalyst (11.09 mg). Therefore, the total adsorption capacity of HZSM-5 (339.89 mg/g_cat._) is much higher than that of Zn/HZSM-5 (110.90 mg/g_cat._), indicating that the introduction of Zn species decreases the adsorption capacity. On the other hand, although there is a larger amount of water adsorption compared to ethylene on both catalysts, this phenomenon is particularly pronounced on the Zn/HZSM-5 catalyst, where the adsorption capacity of water is 15 times higher than that of ethylene. This denotes that the existence of Zn species in the zeolite greatly alters its adsorption properties, reducing the adsorption capacity while enhancing the selective adsorption of water. This selective adsorption, particularly on the Zn/HZSM-5 catalyst, significantly reduces the chances of ethylene coming into contact with active centers, such as acidic centers, thereby decreasing the reaction possibility. This observation also explains the higher ethylene conversion on the HZSM-5 zeolite catalyst compared to the Zn/HZSM-5 zeolite catalyst in the presence of water (Figure 3A). The competitive adsorption of water on ethylene is a crucial factor in the decrease in ethylene conversion in the presence of water. From IR and IGA experiments, it has been found that water adsorption includes both physical and chemical adsorptions and its competitive adsorption with ethylene mainly occurs on the active centers of the zeolite, primarily the acidic centers. Since Zn/ZSM-5 has fewer Brønsted acidic sites (BAS), it intensifies the competitive adsorption behavior between water and ethylene, leading to a significant decrease in ethylene conversion on Zn/HZSM-5 catalysts in the presence of water. Furthermore, the competitive adsorption of water not only decreases the possibility of ethylene reaction and thus reduces the ethylene conversion but also greatly inhibits the adsorption of other intermediate reactant species on the acidic sites and the occurrence of secondary reactions. Since the formation of aromatics requires the processes of ethylene oligomerization, cyclization, dehydrogenation, and hydrogen transfer reactions, the decrease in the adsorption capacity of BAS acidic sites caused by the presence of water also greatly reduces the formation of aromatics and lowers the selectivity of aromatics.

## 3. Experimental

### 3.1. Catalyst Preparation

NaZSM-5 zeolite (Si/Al = 30) was prepared from silica sol, sodium alumina, tetrapropylammonium hydroxide (TPAOH), and deionized water with the molar composition of SiO_2_:0.0167Al_2_O_3_:0.033NaO_2_:0.15TPAOH:30H_2_O. The crystallization was conducted at 170 °C for 48 h under rotation. The solid product was recovered by centrifugation, washing, drying overnight at 100 °C, and then calcining at 560 °C for 13 h. The HZSM-5 zeolite was obtained by ion-exchanging NaZSM-5 zeolite with aqueous NH_4_NO_3_ solution (1 mol/L) two times at 80 °C for 4 h and then calcining at 560 °C for 8 h.

Zn/HZSM-5 with Zn content of 1.8 wt.% was prepared by incipient wet impregnation of HZSM-5 in Zn(NO_3_)_2_ solution at room temperature for 12 h, followed by drying at 100 °C overnight and calcination at 560 °C for 8 h in air.

### 3.2. Catalyst Characterization

Fourier-transform infrared spectroscopy (FTIR): IR studies of the co-adsorption of ethylene and water on Zn/HZSM-5 and HZSM-5 were recorded on a Bruker VERTEX 70 spectrometer (MA, USA) equipped with CaF_2_ optics. Prior to measurement, the catalyst was pretreated in an Ar flow (45 mL/min) at 450 °C for 1 h. After that, the sample cell was cooled down to 30 °C in a flow of Ar, and the background spectrum was recorded. Then, the sample was exposed to a *x*H_2_O/C_2_H_4_/Ar mixture (10 mL/min C_2_H_4_, 45 mL/min Ar) for 0.5 h. H_2_O was continuously introduced into the in situ chamber by flowing Ar (45 mL/min) through a H_2_O bubbling saturator kept at 0 °C or 25 °C. Subsequently, the sample was purged with Ar (45 mL/min) for 1 h to remove the gaseous and physiosorbed C_2_H_4_ and H_2_O. The spectra were recorded with a resolution of 4 cm^−1^ and cumulative 64 scans.

Intelligent gravitation analyzer (IGA): The competitive adsorption of ethylene and water on the zeolite samples was measured using an intelligent gravitation analyzer from Hiden Analytical(Warlington, England). A 20–40 mesh catalyst was loaded into sample cell and pretreated at 300 °C for 2 h under an Ar atmosphere. The sample was then cooled to 30 °C and exposed to ethylene gas and water vapor for saturate adsorption. The samples were thoroughly purged with an argon gas stream to remove physically adsorbed ethylene and excess water. The temperature program desorption was then conducted by heating the sample from 30 to 250 °C at a rate of 4 °C/min while recording the mass spectrum. The sample weight was recorded using buoyancy correction throughout the entire process. In order to completely distinguish between the physical and chemical adsorption of water on the catalyst, the temperature was maintained at 120 °C and 250 °C until there was no further change in the quality of the catalyst.

X-ray absorption fine spectroscopy (XAFS): Zn *K*-edge XAFS was collected at the 1W1B beam line in the Beijing Synchrotron Radiation Facility (BSRF) using a Si (111) double crystal monochromator. The collected XAFS spectra were processed using the Athena program in the Demeter software package (version 0.9.26) [35], and the photon energy was calibrated using the *K*-edge of Zn in metal Zn foil. Athena software was used to perform linear combination fitting (LCF) analysis on the near edge spectrum of 9650–9730 eV energy range. The XANES spectra (μ^exp^ (E)) of each sample were linearly fitted from the XANES spectra (μ_i_^REF^ (E)) of four reference samples. μ^LCF^ (E) = w_1_μ_1_^REF^ (E) + w_2_μ_2_^REF^ (E) + w_3_μ_3_^REF^ (E) + w_4_μ_4_^REF^ (E), w_1_ + w_2_ + w_3_ + w_4_ = 1 [36,37].

The extended X-ray absorption fine structure (EXAFS) spectra were obtained by using a standard procedure of data reduction and least-squares fitting following the IFEFFIT code; the phase and amplitude functions were analyzed using the FEFF 9.0 code. The data used in the EXAFS fitting ranged from k = 2.3 to 11.7 Å^−1^. The fitting was carried out in R space in the range of 1.0–3.5 Å, with a sine window and multiple k*^n^* weighting (*n* = 2). The parameters describing electronic properties (e.g., correction of the photoelectron energy origin) and local structural environment (coordination numbers, CN, bond length, R, and their mean-squared relative derivation, σ^2^) around the absorbing atoms were varied during fitting.

Several typical Zn compounds were selected as the reference samples, as listed in Table 4, to analyze the structure and state of Zn species in zeolites [23]. Reference sample Zn/HZSM-5-IE-0.66%, which is Zn-containing HZSM-5 prepared by ion exchange, represents zinc cations, which has been evidenced as Zn(OH)^+^ species by Pinilla-Herrero et al. [30]. Reference sample Zn/silicalite-1–0.1% with Zn content of 0.1% represents ZnO clusters in the pores of the zeolite, based on the weak interaction between ZnO and silicalite-1, while reference samples Zn/silicalite-1–5% with Zn contents of 5% represent ZnO crystallites on the external surface of the zeolite [38]. Reference sample willemite, with the chemical composition of Zn_2_SiO_4_, has tetrahedral Zn-O species with Zn–O–Si structure [39]. By fitting the EXAFS spectra, the coordination number (CN), interatomic distance (R), Debye–Waller factor (σ^2^), and R-factor of the reference samples are obtained and summarized in Table 4. ZnO shows a pronounced peak of 1.97 Å for the nearest Zn-O scattering, which corresponds to four oxygen neighbors [25]. The Zn species in Zn/silicalite-1–0.1% and Zn/silicalite-1–5% samples also exhibit tetrahedrally coordinated structures [40]. The coordination number of Zn-O is around 6 in Zn/HZSM-5-IE-0.66%, indicating an octahedral structure of Zn-O in zinc cation species with a strong interaction with the acidic hydroxyl of ZSM-5 [21,30].

### 3.3. Catalyst Tests and Analytic Procedures

The ethylene-to-aromatic (ETA) reaction was conducted in a continuous-flow fixed-bed reactor with an inner diameter of 10 mm. The sample was compacted into wafers, crushed, and sieved to 20–40 mesh size prior to use. In a typical run, 1.5 g of the zeolite catalyst was loaded into the reactor and pretreated under a nitrogen flow (50 mL/min) at the desired reaction temperature (470 °C) for 8 h. After the pretreatment, ethylene was introduced into the reactor at a flow rate of 36 mL/min, with a gas weight hourly space velocity (WHSV) of 1.8 h^−1^ and a reaction pressure of 0.1 MPa.

Water or nitrogen gas was introduced into the ethylene aromatization reaction system as an additional reaction atmosphere, with a molar ratio of ethylene to the reaction atmosphere of 3:1, 3:2, and 1:1, corresponding to ethylene contents of 75%, 60%, and 50%, respectively. During these processes, the flow rate of ethylene was maintained at 36 mL/min, while the flow rate of water or ethylene was adjusted accordingly based on the ratio.

The gaseous and liquid products were separated using a cold trap. The gaseous products were analyzed online by an Agilent 7890A gas chromatograph (CA, USA) equipped ith one thermal conductivity detector (TCD), two flame ionization detectors (FID), and three capillary columns (DB-1, OxyPlot, and Al_2_O_3_/KCl). The liquid organic phase was analyzed by another Agilent 7890A gas chromatograph equipped with an FID and a capillary column (HP-PONA).

The ethylene conversion and product selectivity in carbon-based molar percentage were calculated by the following equations:

$$conversion=\frac{F_{inlet}-F_{oulet}}{F_{inlet}}\times 100\%$$

$$Selectivity(i)=\frac{n_{i}F_{i}}{\Sigma n_{i}F_{i}}\times 100\%$$

where *F_inlet_* and *F_outlet_* are the inlet and outlet reactant carbon molar flow rates, respectively, *F_i_* represents the molar flow rate of product *i* with *n_i_* carbon atoms in the effluents, and *Σn_i_F_i_* represents the sum of the carbon molar flow rates of all products in the effluents. The selectivity is expressed as a carbon-based molar percentage.

## 4. Conclusions

The presence and impact of water in the ethylene aromatization reaction show a complex trend. On the one hand, the conversion of ethylene significantly decreases, and the selectivity toward aromatic hydrocarbons and light alkanes also decreases noticeably; on the other hand, the selectivity toward hydrogen increases significantly, indicating that the dehydrogenation pathway in the formation of aromatic hydrocarbons is promoted while the hydrogen transfer pathway is inhibited. Based on this, this study examines the influence of water from the perspectives of active site distribution of catalyst and reactant adsorption characteristics.

The detailed LCF analysis of XANES on fresh and deactivated Zn/HZSM-5 catalysts reveals substantial differences in the state and distribution of Zn species, depending on the reaction atmospheres and water contents. Specifically, it is observed that in the pure ethylene reaction system or nitrogen atmosphere, the zinc species present on the deactivated catalyst predominantly consists of ZnO clusters and crystalline ZnO. However, in the ethylene–water reaction system, the deactivated catalyst retains ZnOH^+^ species as active sites for aromatization dehydrogenation. Notably, the concentration of ZnOH^+^ species increases as the water content in the system increases. When the molar concentration of water in the reaction system reaches 50%, the concentration of ZnOH^+^ species on the deactivated catalyst is similar to that on the fresh catalyst, where the dehydrogenation reaction in the ethylene aromatization process is significantly promoted. It has been widely recognized that ZnOH^+^ plays a crucial role as the primary active site for the dehydrogenation reaction. The notable enhancement of the dehydrogenation reaction in the ethylene aromatization process when water is added can be attributed to the suppression of the transformation of ZnOH^+^ species on the catalyst to ZnO species. This inhibition helps maintain a high dehydrogenation activity by preserving the ZnOH^+^ active sites.

Further investigations using IR and IGA techniques to study the simultaneous adsorption of water and ethylene on HZSM-5 and Zn/HZSM-5 zeolites reveal a competitive adsorption behavior between the two molecules. Specifically, when water is present on the Zn/HZSM-5 catalyst, it greatly impedes the adsorption of ethylene and thus reduces the secondary reaction of olefins and slowing down the rate of carbonaceous species formation, resulting in reduced ethylene conversion and lower aromatic selectivity. However, it has been observed that on the HZSM-5 catalyst, although there is competitive adsorption between water and ethylene, water does not have a significant inhibitory effect on the adsorption of ethylene. This finding explains why the presence of water on the HZSM-5 catalyst still maintains a favorable ethylene conversion.

This study reveals the effects of water on the reactivity and product distribution in ethylene aromatization from both changes in the active center structure and the adsorption of reactant species. Whether it is MTA or light olefin aromatization, the design of the catalyst is based on the requirements of reaction activity and target product selectivity, but little attention is paid to the impact of other products or byproducts in actual application processes. Due to the large amount of water generated during the methanol conversion process, its impact on the light olefin aromatization process cannot be ignored, but existing studies have not paid attention to this aspect. Our work reveals that the presence of water in the reaction process significantly affects the adsorption and reaction activity of ethylene, leading to a decrease in ethylene conversion. From this perspective, increasing the acidic density of the catalyst’s Brønsted acidic sites can promote the adsorption and reaction of ethylene and intermediate species. The presence of water also has its benefits in helping the catalyst maintain the amount of ZnOH^+^ species and excellent dehydrogenation performance. From this perspective, reducing the zinc content appropriately can not only maintain high dehydrogenation performance but also reduce the consumption of Brønsted acidic sites and improve reaction activity and aromatic selectivity. These findings provide valuable insights into the process of aromatization and support the development of efficient aromatization catalysts.

## Figures and Tables

**Table 1:** The effect of water on the performance of HZSM-5 catalyst in the aromatization reaction of ethylene.

Sample	Feed Composition	Conversion (%)	Hydrocarbon Selectivity (C Molar%)				H_2_ Selectivity. (Molar %)
			C_1_^0^–C_4_^0^	C_3_^=^–C_4_^=^	C_5_+	Arom.	
Zn/HZSM-5	ethylene	99.6	31.3	1.1	0.9	66.6	36.1
	C_2_^=^:H_2_O = 3:1	95.9	19.5	9.9	5.3	65.1	49.5
	C_2_^=^:H_2_O = 3:2	87.3	18.6	15. 7	6	59.6	52.2
	C_2_^=^:H_2_O = 1:1	78.1	16	19.9	5.9	58.1	56.1
HZSM-5	ethylene	98.9	48.4	2.0	3.1	46.5	14.2
	C_2_^=^:H_2_O = 3:1	96.3	45.6	10	3.8	40.5	6.2
	C_2_^=^:H_2_O = 3:2	93.9	39.2	18.2	9.5	32.6	5.6
	C_2_^=^:H_2_O = 1:1	85.7	24.9	35.6	14.7	23.9	3.7
	ethylene ^[14]^ (500 °C, TOS 3.2)	86	42	12	/	46	/
	C_2_^=^:H_2_O = 1:3.73 ^[14]^ (500 °C, TOS 3.2)	81	42	14	/	44	/

/: The cited literature does not provide the relevant data.

**Table 2:** Zn *K*-edge XANES and EXAFS fit parameters of Zn/HZSM-5 samples.

Samples	E_0_	Zn *K*-Edge EXAFS Fit Parameters ^a^				
		Contribution	CN	R (Å)	σ^2^	R Factor
Fresh Zn/HZSM-5	9664.43	Zn-O	6.49	2.03	0.013	0.005
Used Zn/HZSM-5 (pure C_2_^=^)	9663.48	Zn-O	3.66	1.97	0.009	0.006
Used Zn/HZSM-5 (C_2_^=^:H_2_O = 3:1)	9663.50	Zn-O	4.04	1.98	0.008	0.004
Used Zn/HZSM-5 (C_2_^=^:H_2_O = 3:2)	9663.76	Zn-O	4.76	1.99	0.010	0.004
Used Zn/HZSM-5 (C_2_^=^:H_2_O = 1:1)	9664.42	Zn-O	6.09	2.03	0.001	0.005
Used Zn/HZSM-5 (C_2_^=^:N_2_ = 3:1)	9663.41	Zn-O	3.74	1.97	0.009	0.005

^a^: CN: coordination number; R: distance between absorber and back scatterer atoms; σ^2^: Debye–Waller factor; R-factor: $\sum_{i}{\left({data}_{i}-{fit}_{i}\right)}^{2}/{\left({data}_{i}\right)}^{2}$.

**Table 3:** The adsorption capacity of water and ethylene on Zn/HZSM-5 and HZSM-5.

Sample	Weight Loss ^a^ (mg)	Chemical Adsorption Capacity ^b^ (mg/g_cat._)	
		H_2_O	C_2_H_4_
Zn/HZSM-5	11.09	103.46	7.44
HZSM-5	33.79	186.59	151.30

^a^ calculated from the weight loss above 120 °C. ^b^ chemical adsorptive quantity of H_2_O and C_2_H_4._

**Table 4:** Zn *K*-edge XANES and EXAFS fit parameters of reference samples.

Samples	Description	E_0_ (eV)	Zn *K*-Edge EXAFS Fit Parameters ^a^					
			Contribution	CN	R (Å)	S_0_^2^	σ^2^	R Factor
Zn/HZSM5-IE-0.66%	ZnOH^+^	9664.1	Zn-O	5.74	2.07	0.91	0.010	0.005
Zn/silicalite-1–0.1%	ZnO clusters	9663.7	Zn-O	3.77	2.02	0.91	0.007	0.010
Zn/silicalite-1–5%	ZnO crystallite	9662.1	Zn-O	3.90	1.97	0.91	0.007	0.005
Willemite	Zn-O-Si structure	9662.5	Zn-O	3.52	1.95	0.91	0.004	0.020

^a^: CN: coordination number; R: distance between absorber and back scatterer atoms; σ^2^: Debye–Waller factor; R-factor: $\sum_{i}{\left({data}_{i}-{fit}_{i}\right)}^{2}/{\left({data}_{i}\right)}^{2}$; S_0_^2^: the amplitude factor, which was fixed at a value of 0.91, forms the model compound.

## Data Availability

Data are contained within the article.
